# Characterizing post-mortem blood concentrations in methadone-related deaths

**DOI:** 10.1007/s00414-025-03620-0

**Published:** 2025-10-08

**Authors:** Guido Pelletti, Valentina Giunchi, Paolo Fais, Mattia Innocenti, Arianna Giorgetti, Elisabetta Poluzzi, Michele Fusaroli, Susi Pelotti

**Affiliations:** 1 Department of Medical and Surgical Sciences, Unit of Legal Medicine, University of Bologna, Via Irnerio 49, 40126 Bologna, Italy; 2 Medicina Legale e Risk Management, Azienda USL di Bologna, Bologna, Italy; 3 Department of Medical and Surgical Sciences, University of Bologna, Bologna, Italy

**Keywords:** Forensic toxicology, Post-mortem, Methadone, Fatal intoxication, Blood concentration

## Abstract

Methadone is a widely used opioid agonist in maintenance therapy for opioid addiction; however, its global diversion is increasing. Determining methadone’s role in drug-related deaths is often challenging due to its pharmacological properties, such as tolerance, and the co-consumption of psychotropic substances. This study aims to identify the demographic, clinical, and therapeutic characteristics of methadone-related deaths to support forensic interpretation. A systematic literature review was conducted to retrieve and analyze publications on methadone-related deaths that included post-mortem toxicological analyses. The data were integrated with methadone-related death cases examined at the Institute of Forensic Medicine in Bologna. The literature review identified 58 articles for data extraction. Aggregated data were available in 29 papers, while individual-level data were reported in 30 studies (with one paper providing both). The aggregated data comprised 3,148 cases, with a median of 80 cases per study (interquartile range, IQR 32–129). A total of 240 individual cases were retrieved from the literature (*n* = 204) and Bologna’s Forensic Medicine records (*n* = 36). Among these, 27 were children, 190 were adults, and 23 had no reported age. The median blood methadone concentration was 300 ng/mL (IQR 195–600 ng/mL) in children and 503 ng/mL (IQR 300–933 ng/mL) in adults, overlapping with levels observed in living patients undergoing methadone maintenance therapy. Statistically significant differences in methadone blood concentrations were found between deaths attributed solely to methadone and those involving multidrug intoxication, with higher concentrations observed in the former group (*p* < 0.001). This review confirms previous findings on the overlap between therapeutic and toxic methadone blood concentrations, highlighting the increasing risk of multidrug intoxications. The extensive number of cases collected and reviewed serves as a valuable resource to assists forensic pathologists in interpreting post-mortem methadone concentrations in relation to the cause of death.

**Supplementary Information:**

The online version contains supplementary material available at 10.1007/s00414-025-03620-0.

## Introduction

Opioid maintenance therapy with methadone is an essential component in treating opioids addiction and reducing related morbidity and mortality [1, 2]. This opioid agonist, when administered in a steady state, prevents withdrawal symptoms without causing euphoria, thus reducing the urge to abuse opioids. The effectiveness of methadone in reducing opioid-related deaths has led to a significant increase in its prescription over the last two decades. Nonetheless, this rise in prescription, together with the undeniable potential for abuse of methadone itself, has, in turn, contributed to the phenomenon of methadone diversion for recreational use, which contributed to several cases of overdose deaths around the world [3, 4].

Post-mortem methadone concentrations detected in cases of fatal methadone intoxication can overlap with those found in living intoxicated individuals or in patients undergoing maintenance therapy [5–7], plausibly due to interindividual variability in methadone tolerance. Habitual users–whether following a maintenance prescription or because of habitual abuse–develop a much higher tolerance than individuals using methadone recreationally (i.e., just as experimental episodes or in socially-endorsed situations), where a lower dose may be sufficient to cause death - particularly when combined with other drugs [6]. Due to this variability, most methadone-related deaths are accidental [7] –whether due to improper prescription, erroneous intake, or illegal abuse– and typically occur during the first few weeks of opioid maintenance therapy, often resulting from rapid dosage escalation and subsequent fatal respiratory depression [8]. Plausibly, also other risk factors concur to methadone-related toxicity and deaths, as coadministration of other drugs [9]. Because of this variability, the medico-legal interpretation of post-mortem toxicological findings in methadone-related fatalities can be challenging if no further information about the case history is available [10].

A large body of literature has investigated post-mortem cases to characterize methadone-related deaths and understand whether there is a toxic or lethal post-mortem concentration that could help both in understanding the toxicity of the molecule and in evaluating the influence of methadone in death for medico-legal purposes.

This study reviews published cases of methadone-related deaths reporting post-mortem methadone concentrations to identify individual characteristics that may assist in the forensic interpretation of these deaths. A series of 36 cases of methadone related deaths from the Legal Medicine of the University of Bologna, Italy were also included in the analysis.

## Materials and methods

### Study design

A systematic literature review retrieved and analyzed all the publications concerning methadone-related deaths reporting post-mortem toxicological analysis. Extracted data were divided into aggregated data and individual-level data. First, we presented global statistics from the complete sample to explore methadone-related deaths’ characteristics. Second, we focused exclusively on individual data to better assess the effect of demographic, anamnestic, and therapeutic variables on the relationship between concentration and mortality. Retrieved data were integrated with 36 methadone-related deaths on which a forensic autopsy was performed at the Institute of Legal Medicine of Bologna, Italy.

### Search strategy

In July 2024, two authors performed the Web-based systematic search of the literature by querying the search engines MEDLINE/PUBMED, searching for the Mesh terms “Methadone” AND “Forensic Toxicology” OR “Death” OR “Poisoning”, and SCOPUS searching for the terms “Methadone” (Article title) AND “Forensic toxicology” (Article title, Abstract, Keywords) OR “Death” (Article title, Abstract, Keywords) OR “Poisoning” (Article title, Abstract, Keywords). The research query was intentionally kept broad to ensure sensitivity and minimize the risk of misleading results. Retrieved articles were then screened to assess their eligibility. Two reviewers independently screened the titles and abstracts first, followed by a review of the full text. To identify relevant reports on methadone-related deaths for this review, we assessed whether the following eligibility criteria were met.

- Type of study: original articles, case-reports and case-series published between January 2000 and July 2024.
- Papers reporting “methadone related deaths”, namely fatalities where toxicological post-mortem analysis revealed the presence of methadone in post-mortem blood.
- Data on methadone concentration range and cause of death.

Non-English language studies were excluded. A comprehensive database of the retrieved articles was created and checked for removing duplicates (including studies conducted on the same database).

### Information retrieval

The following data were retrieved from included papers using a pre-piloted Excel sheet. The papers were categorized into two groups: studies reporting only aggregated data and those providing data on individual deaths.

The following information were extracted.

- Bibliographic data: author(s), year of publication, country where the study was performed.
- Type of study: retrospective study (based on autopsy report o registry), case report/series.
- Demographic data about the victim(s): sex (male/female), age.
- Cause of death, divided into “acute methadone intoxication” and “multidrug intoxication” based on the detection of relevant concentration of other psychoactive drugs (illicit drugs or medications) in the blood. For this categorization we relied on the interpretation of the paper’s author, and in case of doubts, three reviewers evaluated the case based on circumstantial data, autopsy findings, and the concentrations of all detected substances.
- Methadone maintenance therapy (MMT): considering the well-known inter-individual tolerance to methadone, it was investigated whether the deceased was undergoing MMT (yes/no/unknown). The posology was retrieved when available.
- History of addiction: present, absent, unknown.
- Methadone blood and urine concentration (ng/ml).
- Other psychoactive drugs detected in post-mortem toxicological analyses and their concentrations (ng/ml). The drugs were also classified into categories based on their potential pharmacokinetic or pharmacodynamic interaction with methadone, in accordance with Drugbank [11]: CYP2B6 inducer, CYP3A4 inducer, CYP3A4 inhibitor, Central Nervous System (CNS) depressant (or depressant agents), QT prolonger, other concomitant medications not included in the previous categories.

### Statistical analysis

Data from children and adults were analyzed separately, due to different body composition, volume of distribution and administration method. In fact, voluntary intake is highly unlikely in children, making voluntary administration or accidental ingestion more probable. Specifically, children were defined as individuals under 7 years of age, as no data were collected for individuals between the ages of 7 and adulthood. Data on children were analyzed using a descriptive analysis of the collected variables. Categorical variables were described as absolute and percentage frequencies, while continuous variables were summarized using the median, interquartile range, minimum and maximum values. For adults, both aggregated and individual-level data were analyzed using descriptive statistics. For individual data, further analyses were conducted, including the study of blood methadone concentrations and their characterization by sex, MMT (yes or no – data with unknown status were excluded), and cause of death. These analyses involved plotting the distribution of blood concentration by group, applying the Wilcoxon-Mann-Whitney test for assessing differences in blood concentrations between group, and performing a regression analysis to determine whether methadone blood concentrations could be influenced by the coadministration of other drugs with potential pharmacokinetic or pharmacodynamic interactions with methadone. All analyses were performed using R v.4.3.1.

## Results

**Fig. 1:**
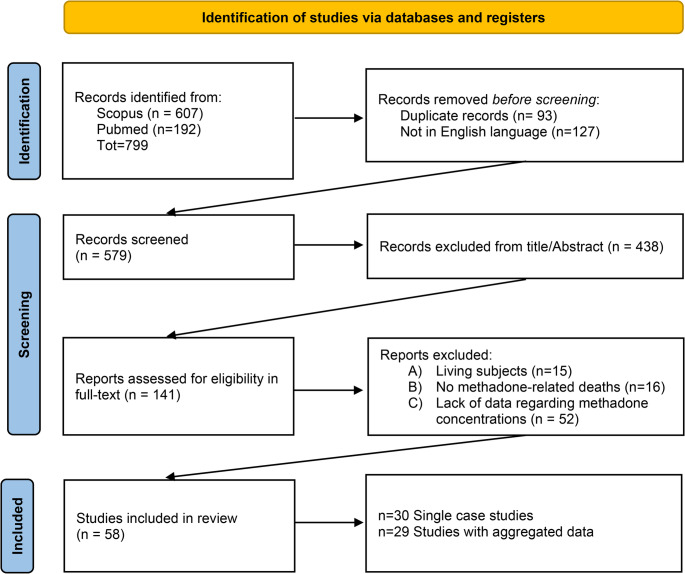
Selection process of the article to include in the review. Aggregated data were available in 29 papers, while individual-level data were reported in 30 studies (with one paper providing both)

The literature review led to the selection of 58 papers [3, 6, 7, 9, 10, 12–64] for data extraction. Aggregated information was available in 29 papers [3, 7, 10, 12–37], while individual-level data were reported in 30 papers [6, 9, 36, 38–64] (Fig. 1). One paper reported data in both individual and aggregated form [36].

Aggregated data (Table S1) reported a total sample size of 3,148 cases, with a median of 80 cases per study (interquartile range, IQR 32–129). Among studies that specified sex, the total number of males was 2,087 (77.3%) and females 613 (22.7%), with a median number of 61 males (range 25–105) and 21 females (range 6–42) per study. The median age was 36 years (age range 17–57). Additionally, the cause of death was acute methadone intoxication in 1,941 (61.7%) cases and multidrug intoxication in 893 (28.4%).

A total of 240 single cases were retrieved from the literature (Table S2), including the 36 cases from the Institute of Legal Medicine of Bologna (Table S3). In total, 27 were children, 190 were adults, and 23 did not report an age. The latter were assumed to be adults from the analysis of the data. Table 1 reports data on children’s cases, while Table 2 reports data on adult cases.

**Table 1:** Descriptive analysis of children cases. IQR: interquartile range

Variable	*N* = 27
Sex	
Female	7 (26.9%)
Male	19 (73.1%)
Unknown	1
Age	
Median (IQR) [range]	0.92 (0.08-2) [0–6]
Country	
United States	8 (29.6%)
Italy	5 (18.5%)
United Kingdom	5 (18.5%)
France	4 (14.8%)
Canada	2 (7.4%)
Poland	1 (3.7%)
Slovakia	1 (3.7%)
Australia	1 (3.7%)
Cause of death	
Methadone intoxication	23 (85.2%)
Multidrug	4 (14.8%)
Methadone blood conc (ng/ml)	
Median (IQR) [range]	300 (195–600) [26–900]

**Table 2:** Descriptive analysis of adult cases (> 21 years). IQR: interquartile range. MMT: methadone maintenance treatment

Variable	*N* = 213
Sex	
Female	42 (21.7%)
Male	152 (78.4%)
Unknown	19
Age	
Median (IQR) [range]	36 (29–44) [16–64]
Unknown	23
Country	
Italy	112 (52.6%)
Germany	50 (23.5%)
United Kingdom	17 (8.0%)
Poland	16 (7.5%)
Switzerland	8 (3.8%)
United States	4 (1.9%)
France	1 (0.5%)
Cause of death	
Methadone intoxication	109 (51.2%)
Multidrug	104 (48.8%)
Methadone blood conc (ng/ml)	
Median (IQR) [range]	503 (300–933) [2–11,580]
MMT	
No	22 (15.4%)
Yes	121 (84.6%)
Unknown	70
Posology (mg/die)	
Median (IQR) [range]	45 (40–80) [10, 720]
Unknown	175

**Fig. 2:**
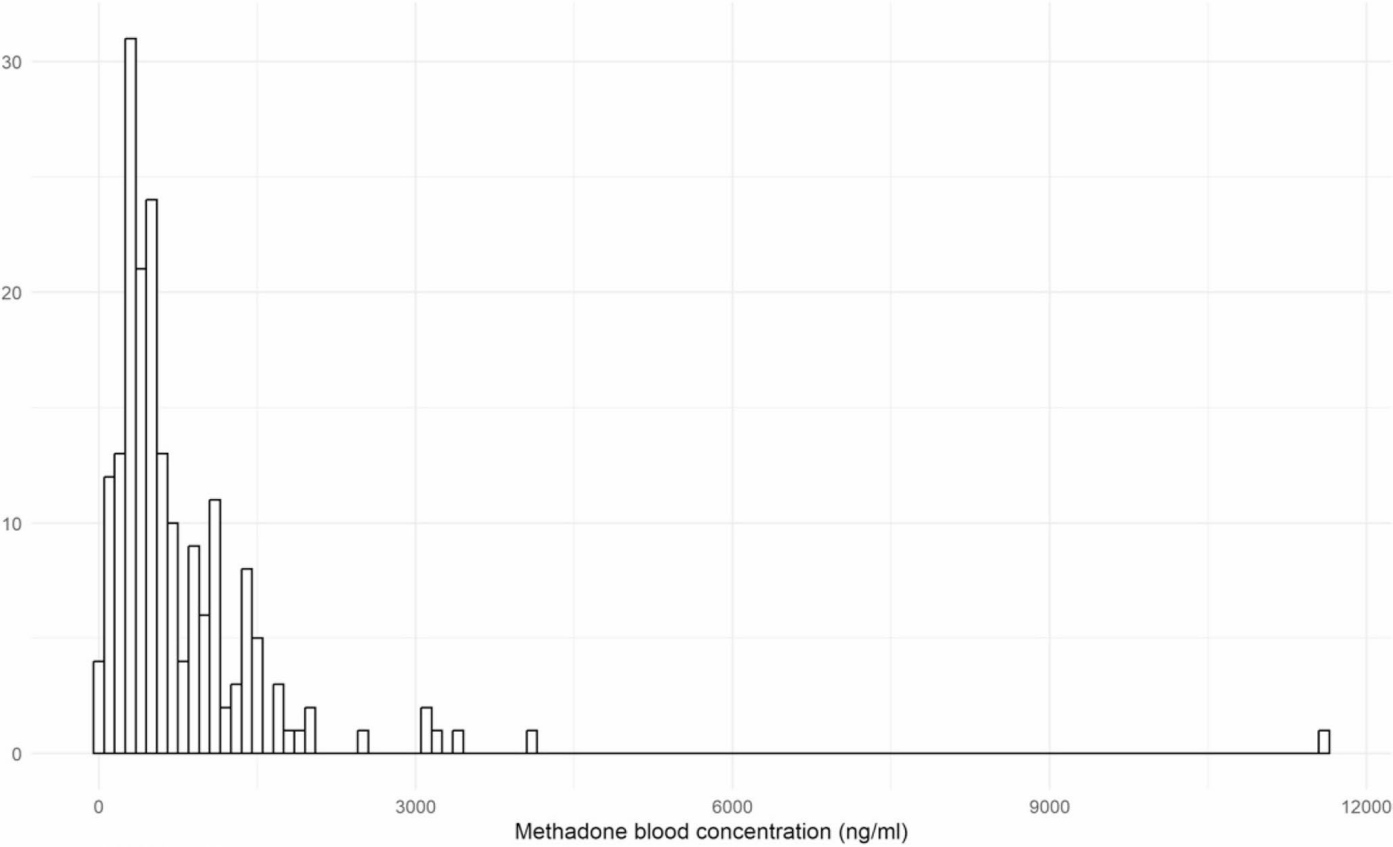
Distribution of methadone blood concentration in all single cases. Total number of cases: 240. Cases with available methadone blood concentration: 224

Most of the children’s cases involved neonates or infants (median age 0.92 years, IQR 0.08–2 years), with males comprising 73.1% of the cases. Among children, in 3 cases the mother was in MMT, and only in 4 cases a multidrug intoxication was observed. There were 213 adult cases, with 78% being males. The median age was 36 years (IQR 29–44). Most cases (53%) were from Italy, also due to the inclusion of data from Bologna’s Forensic Medicine Institute. Additionally, 23% of cases were from Germany, and 8% from the United Kingdom. Cases were nearly equally distributed between “methadone intoxications” and “multidrug intoxications” (51% and 49%, respectively). The median methadone blood concentration was 503 ng/ml (IQR 300–933), with distribution reported in Fig. 2. Twenty-two subjects were on MMT, 84.6% of the subjects for whom the data was known, as this information was unavailable for 70 cases. Comparison of methadone blood concentration in male and female subjects (Table S4 and Figure S1), in deceased with and without MMT (Table S4 and Figure S2) and in methadone intoxication and multidrug intoxication (Table S4 and FigureS3) showed statistically significant differences only between deaths caused by methadone and multidrug intoxications, with higher concentrations in the former group (*p* < 0.001). Regression analysis showed no significant association between drugs with potential pharmacokinetic/pharmacodynamic interactions and methadone blood concentrations (Table S5).

## Discussion

A large body of research has investigated, in the past 24 years, post-mortem blood concentrations in methadone-related fatalities, characterizing both acute methadone intoxication and multidrug intoxications. We divided studies fulfilling the inclusion criteria, into two categories: studies reporting aggregated data (Table S1) and studies reporting single cases (Table S2), that were integrated with cases from the Institute of Legal Medicine of Bologna (Table S3).

The analysis of studies reporting aggregated data has shown higher post-mortem methadone blood concentrations in women compared to men [18, 29], in subjects on MMT [10] and in cases of multidrug intoxication [21]. However, these results are not statistically significant and are similar to those observed in living subjects [24]. Further, these studies often involve limited populations, focusing on a single center or having different research objectives, with poor details on post-mortem blood concentration. For this reason, it became necessary to extract data to assess the influence of individual variables.

The analysis of studies reporting individual data was performed separately for children (< 7 years) and adults (> 19 years), as there are significant physiological differences that may influence both the analysis and interpretation or toxicological results.

Most of the children fatalities concerned acute methadone intoxication (85.2%). Intoxications in children mainly occurred as a consequence of accidental ingestion or administration by third parties of methadone in syrup form [47, 48]. Poisoning through ingestion via breast milk is a topic widely addressed in the literature, as maternal methadone use is not, per se, a contraindication to breastfeeding. This is primarily because there are no clear reports in the literature of fatal intoxication attributable to methadone exposure through breast milk [65]. Of particular interest, the study by Madadi et al. [48] reported two infant fatalities whose mothers were receiving methadone maintenance therapy. Both infants were heterozygous for the reduced activity form of the P-glycoprotein transporter, and one also carried a CYP2B6 pharmacogenetic variant thought to decrease methadone metabolism. However, the authors explicitly state that both infants had other contributing factors that may have led to their deaths, and that it would be inappropriate to conclude that methadone exposure via breast milk was the sole cause of death in these cases. In our review of fatal cases, we observed a median blood concentration in children of 300 ng/mL (IQR 195–600 ng/mL), and urine analyses were rarely conducted. For the purposes of data interpretation, our analysis focused on blood concentrations, as urinary levels are influenced by factors such as water balance, fluid intake, perspiration, renal insufficiency, and the timing of the last bladder voiding. Consequently, urinary concentrations are of limited value in determining the cause of death. Notably, in children under one year of age, acute methadone intoxication has been described even with concentrations below 100 ng/mL. The results obtained were similar to those observed in systematic review of 38 studies on methadone toxicity, including 29 pediatric cases of fatal methadone ingestion, found an average concentration of 385 ng/mL with a range of fatal concentrations starting from 60 ng/mL [66]. Therefore, concentrations considered “therapeutic” in adults can cause fatal acute poisoning in children, even in the absence of concomitant medications.

The analysis of single case studies involving adults included 190 fatal intoxications, mostly males (78.4%), with an average age of 36 years (IQR 29–44). Contrarily to what was described in previous single-center studies, methadone blood concentrations observed in our review were not significantly different between men and women, nor between individuals who were or were not in MMT. However, we found a significant difference between individuals who died from acute methadone intoxication (median 630 ng/ml; mean 994 ng/ml) and those who died from multidrug consumption (median 399 ng/ml; mean 524 ng/ml). This suggests, as described in previous studies, that co-consumption of other legal or illegal drugs lowers the lethal threshold of methadone. However, this finding will need to be further investigated by quantitatively analyzing the other detected substances to determine whether methadone played a primary role in the death or if it was caused by other compounds. The increased susceptibility when consumed with other substances could be due to their interaction with the metabolism of the molecule. The pathophysiological explanation is that the concurrent misuse of alcohol or benzodiazepines with methadone significantly increases the risk of central nervous system depression, particularly by enhancing sedation and respiratory depression. Acute alcohol intake has been shown to reduce methadone metabolism by competing for hepatic metabolic enzymes, whereas chronic alcohol consumption induces these enzymes, potentially accelerating methadone clearance and precipitating opioid withdrawal [67]. The synergistic effect of these substances causes a reduction in ventilation and arterial oxygenation, increased arterial carbon dioxide tension, and hypoxia, ultimately leading to death from acute respiratory failure [3, 20].

An interesting finding highlighted by our study pertains to differences in patterns of multidrug intoxication between children and adults. Specifically, while multiple drugs were detected in 48.8% of adult cases, methadone was the sole substance identified in the majority of children cases (85.2%). One possible explanation could be that adults often present with multiple comorbidities, including psychiatric disorders or substance or alcohol use disorders, which involve drugs that interact with the pharmacokinetics and pharmacodynamics of methadone. In our study, linear regression analysis for evaluating drugs interacting with inducers or inhibitors of Cytochrome P450 enzymes showed no differences, likely due to the analysis of a limited group of enzymes and the lack of quantitative data on individual drugs, which were not available.

However, when interpreting post-mortem methadone blood concentrations, it is essential to also consider that methadone can cause QT interval prolongation, potentially leading to severe arrhythmias such as torsade de pointes. This risk is heightened when methadone is co-administered with other QT-prolonging substances or with drugs that interfere with hepatic metabolism (e.g., CYP3A4 inhibitors) [68]. Medications known to prolong the QT interval include certain antiarrhythmics, antipsychotics, antidepressants, macrolide antibiotics, fluoroquinolones, and antihistamines [69]. Concomitant use of these agents with methadone may significantly increase the risk of life-threatening arrhythmias. Therefore, in cases of methadone-related death, toxicological and clinical assessments should take into account not only post-mortem methadone concentrations but also the potential for pharmacological interactions with these drug classes.

We observed that the average blood concentration of methadone found in adult cases is 503 ng/mL, higher than observed in children, and overlapping with the range of concentration normally reported in living patients undergoing MMT (400–1000 ng/ml). This complicates the forensic analysis of methadone-related deaths, as MMT is now a well-established therapeutic strategy for treating opioid addiction and is generally considered a safe medication. However, the phenomenon of methadone-related deaths among individuals enrolled in methadone maintenance programs is well documented in the literature.

In the cases from the Institute of Legal Medicine of Bologna, most of adult subjects were undergoing MMT. The prevalence is significantly higher than those reported in studies conducted in single-center studies from USA [70, 71]. No association has been demonstrated between the blood concentrations of subjects in MMT and those of naive users or individuals with no known history of use. This data shows that the well-known tolerance to methadone observed in individuals undergoing MMT does not correspond to an increased post-mortem concentration of the drug in cases of death caused by acute intoxication in these subjects, as observed in previous single-center studies [10, 41].

The overlap between potentially fatal concentrations detected post-mortem and therapeutic concentrations observed in living subjects precludes the determination, based solely on the post-mortem blood concentration, of whether the individual died due to a fatal methadone concentration.

Actually, this review provides limited information regarding the enantiomeric form of methadone in fatal cases. Methadone is typically administered as a 50:50 racemic mixture of (R)- and (S)-stereoisomers, with (R)-methadone exhibiting approximately tenfold higher affinity and potency for the µ-opioid receptor compared to the (S)-isomer [11]. The latter lacks significant respiratory depressant effects but retains antitussive properties. In reports of fatal methadone intoxication where the enantiomeric form is not specified, the reference is generally to the racemic mixture. Data typically pertain to racemic methadone unless the enantiomeric composition is explicitly indicated [72]– [73]. Only in particular circumstances—such as treatment with enantiopure L-methadone—are concentrations specific to a single enantiomer analyzed or reported. Consequently, in fatal intoxications where the enantiomeric form is unspecified, toxicological data, concentration ranges, and clinical-legal assessments—including thresholds relevant to the risk of death—refer to racemic methadone.

In conclusion, our study highlights a great heterogeneity in the reports of methadone-related deaths and deaths involving methadone, making it difficult to identify demographic susceptibilities or a concentration cut-off beyond which death from methadone is considered likely. This is because a range of individual factors are probably involved, making it extremely challenging to define an exhaustive model that allows to predict fatality of methadone intoxication based on blood concentration and risk factors. This underscores the importance of a comprehensive forensic approach for each individual case, which considers clinical, circumstantial, medical, autopsy, and toxicological data to allow for a full evaluation of the case and to determine whether the death was caused by methadone or if methadone may have played a role.

## Conclusion

To gain further insights into methadone toxicity and the interpretation of lethal blood concentrations for clinical and forensic purposes, it will be necessary to expand the body of literature by systematically reporting the key variables that can influence methadone toxicity.

## Supplementary Information

Below is the link to the electronic supplementary material.

Supplementary Material 1 (DOCX 391 KB)

## Data Availability

Not applicable.
