# Supplementary material for: Characterizing post-mortem blood concentrations in methadone-related deaths
Source: Int J Legal Med. 2025 Oct 8;140(1):207–16. doi: 10.1007/s00414-025-03620-0 (PMC12808249; doi:10.1007/s00414-025-03620-0)
Supplement: Supplementary file 1 — Supplementary Material 1 (DOCX 391 KB) [file 414_2025_3620_MOESM1_ESM.docx]

**Supplementary material for the manuscript “Characterizing post-mortem blood concentrations in methadone-related deaths”**

**Table S1.** Studies reporting aggregated data. N: number of cases. NA: Not Available. MMT: Methadone Maintenance Treatment.

| Authors | Year | Type of Study | Sample Size | Sex | Age (Range)  *and other info if available* | Country | Cause of death (N) | MMT (N if Available) | Methadone Blood Concentration (ng/ml)* (Mean and Range) |
| --- | --- | --- | --- | --- | --- | --- | --- | --- | --- |
| Fugelstad A [12] | 2021 | Retrospective study (autopsy reports) | 269 | 40 F; 229 M | (15-29) | Sweden | Methadone intoxication (38) | 197 | Mean: 300 (50 - 4100) |
| Iwersen-Bergmann S [10] | 2021 | Retrospective study (autopsy reports) | 107 | 28 F; 79 M | mean 41 (21-65) | Germany | Methadone intoxication (107) | 24 | All cases - Mean: 740 (60-11500); MMT - Mean: 980 (150-2500); NO MMT - Mean: 470 (60-1080) |
| Nicolakis J [13] | 2020 | Retrospective study (autopsy reports) | 23 | NA | NA | Austria | Methadone intoxication (23) | NA | Mean: 860 (60 - 5600) |
| Lusetti M [14] | 2016 | Retrospective study (autopsy reports) | 20 | 20 M | mean 34 | Italy | Methadone intoxication (20) | Y | Mean: 250 (200 - 400) |
|  |  |  | 20 | 20 M | mean 37 | Italy | Methadone intoxication (20) | NA | Mean: 450 (300 - 850) |
| Holm KMD [15] | 2015 | Retrospective study (autopsy reports) | 87 | NA | mean 43 (20-69) | Denmark | Methadone intoxication (61); multidrug (26) | NA | Methadone intoxication - Mean: 800 (160-7600); multidrug - mean: 290 (100-1600) |
| Nielsen MK [16] | 2015 | Retrospective study (autopsy reports) | 99 | 75 M | mean 42 (20-68) | Denmark | Methadone intoxication (71); multidrug (28) | NA | All cases - Mean: 520 (15-5300); methadone intoxication - Mean: 550 (98-5300); multidrug - Mean: 400 (15-2100) |
| Mijatović V [17] | 2014 | Retrospective study (autopsy reports) | 31 | 6 F; 25 M | mean 25 (20-38) | Serbia | Methadone intoxication (5); multidrug (26) | 29 | Methadone intoxication - Mean: 150 (140-170); multidrug - Mean 80 (20-210) |
| Van Den Broecke SML [18] | 2014 | Retrospective study (autopsy reports) | 37 | NA | mean 30 (18-51) | Belgium | Methadone intoxication (1); multidrug (36) | NA | All cases - Mean 810 (100-4130); F - Mean: 850; M - Mean: 640 |
| Bernard JP [3] | 2013 | Retrospective study (autopsy reports) | 264 | 55 F; 209 M | mean 36 | Norway | Methadone intoxication (264) | 69 | Mean: 400 (100-6500) |
| Jantos R [19] | 2013 | Retrospective study (autopsy reports) | 16 | 5 F; 11 M | mean 31 (23-43) | Germany | Methadone intoxication (16) | 8 | (225-3271) |
| Pilgrim JL [20] | 2013 | Retrospective registry study | 206 | 67 F; 139 M | mean 31 (17-51) | Australia | Methadone intoxication (137); Other (69) | 123 | Mean: 600 (100-300) |
| Wunsch M [21] | 2013 | Retrospective study (autopsy reports) | 246 | 61 F; 185 M | mean 36.9 (25-55) | United States | Methadone intoxication (18); multidrug (228) | 58 | Mean: 495 |
| Eiden C [22] | 2012 | Retrospective study (autopsy reports) | 59 | 10 F; 49 M | mean 33 (4 - 49) | France | Methadone intoxication (28); multidrug (31) | NA | Mean: 320 (1 - 2800) |
| Häkkinen M [23] | 2012 | Retrospective study (autopsy reports) | 48 | NA | mean 30 (14-44) | Finland | Methadone intoxication (48) | NA | Mean: 350 (70-2000) |
| Jones AW [24] | 2012 | Retrospective study (autopsy reports) | 503 | 85 F; 418 M | mean 38 (20-75) | Sweden | Methadone intoxication (418); multidrug (85) | NA | Methadone intoxication - Mean: 510; multidrug - Mean: 460 |
| Madden ME [25] | 2011 | Retrospective study (autopsy reports) | 76 | 21 F; 55 M | mean 36 (16-74) | United States | Methadone intoxication (76) | 2 | Mean: 457 (50-3793) |
| Albion C [26] | 2010 | Retrospective study (autopsy reports) | 45 | 13 F; 32 M | mean F: 44 (28-54); M: 43 (20-64) | Canada | Methadone intoxication (11); Multidrug (25); Other (9) | 13 | MMT: 1100; no MMT: 570; Methadone intoxication - Mean: 720 (200-3000); multidrug - Mean: 1220 (traces- 6000) |
| Buchard A [27] | 2010 | Retrospective study (autopsy reports) | 90 | 21 F; 69 M | (19–63) | Denmark | Methadone intoxication (5); multidrug (72); other (13) | NA | Mean: 620 (11 - 8000) |
| Laberke PJ [28] | 2010 | Retrospective study (autopsy reports) | 146 | 44 F; 102 M | mean F: 37 (17-61); M: 36 (17-59) | Switzerland | Methadone intoxication (16); Multidrug (111); Other (19) | 57 | Mean: 1324 (70 - 14000) |
| Paulozzi LJ [29] | 2009 | Retrospective registry study | 87 | 26 F; 61 M | mean 34 (18-60) | United States | Methadone intoxication (87) | 10 | F - Mean: 630; M - Mean: 480 |
| Chugh SS [30] | 2008 | Retrospective study (autopsy reports) | 22 | 7 F; 15 M | mean 37 (27-47) | United States | Methadone intoxication (22) | 3 | Mean: 480 (100 - 900) |
| Shields LB [31] | 2007 | Retrospective registry study | 176 | 69 F; 107 M | mean 38 (17-38) | United States | Methadone intoxication (176) |  | (20-400) |
| Caplehorn JR [32] | 2002 | Retrospective study (autopsy reports) | 57 | NA | NA | Australia | Methadone intoxication (14), multidrug (43) | 41 | Mean: 530 (traces - 5500) |
| Caplehorn JR [33] | 2002 | Retrospective study (autopsy reports) | 31 | 6 F; 25 M | NA | Australia | Methadone intoxication | 26 | Mean: 600 (400 - 740) |
| Ernst E [34] | 2002 | Retrospective study (autopsy reports) | 84 | 27 F; 57 M | mean: F 31.7; M 31.4 | Australia | Methadone intoxication (4), multidrug (50), other (30) | 36 | Mean: 420 (20-1100) |
| Milroy CM [35] | 2000 | Retrospective study (autopsy reports) | 106 | 17 F; 89 M | mean 27.4 | United Kingdom | Methadone intoxication (50); multidrug (56) | NA | Methadone intoxication - Mean 584 (84-2700); multidrug - Mean: 576 (49-2440) |
| Perret G [36] | 2000 | Retrospective study (autopsy reports) | 36 | NA | mean 27 (16-58) | Switzerland | Methadone intoxication (21);  multidrug (15) | 14 | MMT - Mean 928; NO MMT - Mean: 523;  Methadone intoxication - Mean 874; multidrug - Mean: 529; other - Mean: 512. |
| Karch SB [7] | 2000 | Retrospective study (autopsy reports) | 21 | 5 F; 16 M | mean 46.3 | United States | Methadone intoxication (21) | NA | 957 |
| Heinemann A [37] | 2000 | Retrospective registry study | 136 | NA | NA | Germany | Methadone intoxication (132) | 53 | MMT - Mean: 620 (90-2000); NO MMT - Mean: 430 (500-3500) |

*The blood concentration are distinguished according to the study in the following categories: MMT/NO MMT (patients under methadone maintenance therapy or not); Methadone intoxication/multidrug.

**Table S2.** Studies reporting single cases. N: number of cases. MMT: methadone maintenance therapy. NA: Not Available.

| Authors | Year | Type Of Study | Sex | Age | Country | Cause Of Death | MMT | Posology  (Mg/Die) | Methadone Blood Concentration (ng/ml) | Methadone Urinary Concentration (ng/ml) | Concomitants  (ng/ml in Blood) |
| --- | --- | --- | --- | --- | --- | --- | --- | --- | --- | --- | --- |
| Chighine A [38] | 2022 | Case report | M | 0 | Italy | Methadone intoxication | N | NA | 570 | NA |  |
| Flanagan RJ [39] | 2022 | Retrospective study (autopsy reports) | M | 36 | United Kingdom | Methadone intoxication | Y | 50 | 420 | NA | chlordiazepoxide (140); demoxepam (670); nordiazepam (130) |
|  |  |  | M | 27 |  | Methadone intoxication | Y | 30 | 460 | NA | chlordiazepoxide; ibuprofen; olanzapine; promethazine |
|  |  |  | M | 28 |  | Methadone intoxication | Y | 40 | 270 | NA | dihydrocodeine (360) |
|  |  |  | M | 22 |  | Methadone intoxication | Y | 40 | 240 | NA |  |
|  |  |  | M | 32 |  | Multidrug | Y | 40 | 360 | NA | O-desmethylvenlafaxine (910); diazepam (150); nordiazepam (260); venlafaxine (1470) |
|  |  |  | M | 36 |  | Methadone intoxication | Y | 20 | 160 | NA | diazepam |
|  |  |  | F | 31 |  | Multidrug | Y | 10 | 170 | NA | chlordiazepoxide; codeine (1070); diazepam; morphine (10) |
|  |  |  | M | 34 |  | Multidrug | Y | 35 | 280 | NA | chlordiazepoxide (200); demoxepam (760); nordiazepam (170) |
|  |  |  | M | 46 |  | Methadone intoxication | Y | 30 | 250 | NA |  |
|  |  |  | F | 30 |  | Methadone intoxication | Y | 40 | 530 | NA | amisulpride (160); pregabalin (6000); quetiapine (160) |
|  |  |  | M | 49 |  | Methadone intoxication | Y | 78 | 1400 | NA | - |
|  |  |  | M | 35 |  | Methadone intoxication | Y | 60 | 350 | NA | gabapentin (1000); mirtazapine (25); promethazine (260) |
|  |  |  | M | 39 |  | Methadone intoxication | Y | 110 | 430 | NA | chlordiazepoxide; mirtazapine |
|  |  |  | M | 41 |  | Methadone intoxication | Y | NA | 680 | NA |  |
|  |  |  | F | 33 |  | Methadone intoxication | Y | 40 | 420 | NA | diazepam (70); promethazine |
|  |  |  | M | 52 |  | Methadone intoxication | Y | 40 | 640 | NA | diazepam (10); nordiazepam (240) |
|  |  |  | M | 31 |  | Methadone intoxication | Y | 100 | 1040 | NA | mirtazapine (190); normirtazapine (80); pregabalin (7600); quetiapine (10); zopiclone (20) |
| Andersen FD [40] | 2021 | Case report | F | 34 | Denmark | Multidrug | Y | NA | 1100 | NA | quetiapine (500); delta-9-THC (61) |
|  |  |  | F | 52 |  | Methadone intoxication | NA | NA | 900 | NA | quetiapine (7300) |
|  |  |  | F | 49 |  | Multidrug | NA | NA | 270 | NA | ethanol (0.94 g/L); morphine (140); naloxone (10); pregabalin (5200); quetiapine (6900); delta-9-THC (2.5) |
|  |  |  | F | 43 |  | Multidrug | NA | NA | 310 | NA | bupivacaine (520); hydromorphone (20); morphine (1800); quetiapine (260); venlafaxine (760) |
| D’Errico S [6] | 2021 | Retrospective study (autopsy reports) | M | 47 | Italy | Methadone intoxication | Y | NA | 1500 | 10800 | ethanol (1.87 g/L) |
|  |  |  | M | 46 |  | Methadone intoxication | Y | NA | 800 | 5.7 | NA |
|  |  |  | M | 34 |  | Multidrug | Y | NA | 284 | NA | benzoylecgonine (3187); cocaine (71); ethanol (1.44 g/L); morphine (237) |
|  |  |  | M | 33 |  | Multidrug | Y | NA | 1380 | NA | cocaine (1480) |
|  |  |  | M | 46 |  | Multidrug | Y | NA | 882 | NA | benzoylecgonine (604); cocaine |
|  |  |  | M | 28 |  | Methadone intoxication | Y | NA | 4058.53 | NA | eddp (361.1) |
|  |  |  | M | 49 |  | Methadone intoxication | Y | NA | 1137 | NA | eddp |
|  |  |  | M | 55 |  | Methadone intoxication | Y | NA | 1715 | 1341 | ethanol (0.5 g/L); eddp (389) |
|  |  |  | M | 49 |  | Methadone intoxication | Y | NA | 771.07 | NA | eddp (219.03); phenobarbital (45.65) |
|  |  |  | M | 32 |  | Methadone intoxication | Y | NA | 890 | NA | eddp (65) |
|  |  |  | F | 36 |  | Methadone intoxication | N | NA | 407 | NA | 7-aminoclonazepam (543); ethanol (0.63 g/L) |
|  |  |  | M | 43 |  | Methadone intoxication | N | NA | 405 | NA | benzoylecgonine (198); venlafaxine (127) |
|  |  |  | M | 47 |  | Methadone intoxication | N | NA | 333 | NA | NA |
|  |  |  | M | 33 |  | Methadone intoxication | N | NA | 480 | NA | benzoylecgonine (22.1); clonazepam (150); eddp (80); delta-9-THC (1.2) |
|  |  |  | F | 45 |  | Methadone intoxication | N | NA | 307 | NA | quetiapine (639); diazepam (212) |
|  |  |  | F | 45 |  | Methadone intoxication | N | NA | 547 | NA | ethanol (1.19 g/L); eddp (50) |
|  |  |  | M | 36 |  | Multidrug | N | NA | 1470 | NA | benzoylecgonine (6020); cocaine (3120) |
|  |  |  | M | 37 |  | Multidrug | N | NA | 398 | 333 | benzoylecgonine (3187); cocaine |
|  |  |  | M | 43 |  | Methadone intoxication | N | NA | 181 | NA | NA |
|  |  |  | M | 32 |  | Methadone intoxication | N | NA | 1312 | NA | NA |
|  |  |  | F | 46 |  | Methadone intoxication | N | NA | 1700 | 103 | NA |
|  |  |  | M | 45 |  | Methadone intoxication | N | NA | 1830 | 11157 | NA |
|  |  |  | M | 50 |  | Methadone intoxication | N | NA | 210 | NA | ethanol (1.12 g/L) |
|  |  |  | M | 33 |  | Methadone intoxication | N | NA | 469 | NA | ethanol (0.6 g/L) |
|  |  |  | M | 36 |  | Multidrug | N | NA | 972 | 303 | benzoylecgonine (457); eddp (62); ethanol (1.81 g/L); morphine (161) |
| Favia M [41] | 2021 | Retrospective study (autopsy reports) | NA | NA |  | Methadone intoxication | Y | NA | 785 | NA | NA |
|  |  |  | NA | NA |  | Methadone intoxication | Y | NA | 1356 | NA |  |
|  |  |  | NA | NA |  | Methadone intoxication | Y | NA | 626 | NA |  |
|  |  |  | NA | NA |  | Methadone intoxication | Y | NA | 983 | NA |  |
|  |  |  | NA | NA |  | Methadone intoxication | Y | NA | 378 | NA |  |
|  |  |  | NA | NA |  | Methadone intoxication | Y | NA | 4643 | NA |  |
| Nowak K [42] | 2021 | Retrospective study (autopsy reports) | F | 21 | Poland | Methadone intoxication | NA | NA | 578 | 2890 | eddp (57.3); ethanol (0.27 g/L) |
|  |  |  | M | 40 |  | Methadone intoxication | NA | NA | 1180 | >5000 | 7-aminoclonazepam (233); clonazepam (2.8); eddp (104) |
|  |  |  | M | 34 |  | Multidrug | NA | NA | 699 | >5000 | 7-aminoclonazepam (124); cetirizine (6.0); eddp (88.9); hydroxyzine (2.3) |
|  |  |  | M | 46 |  | Multidrug | NA | NA | 449 | 2940 | 7-aminoclonazepam (53.3); amphetamine (36.9); eddp (180); |
|  |  |  | M | 35 |  | Multidrug | NA | NA | 2.4 | 36.7 | ethanol (0.12 g/L); fentanyl (55.4) |
|  |  |  | F | 30 |  | Multidrug | NA | NA | 331 | >5000 | 7-aminoclonazepam (42); codeine (23); n-desmethyl dextromethorphan (20); n-desmethyl dextrorphan (60); dextromethorphan (370); dextrorphan (242); eddp (157); morphine (22); normorphine (532); pseudoephedrine (17.2) |
|  |  |  | M | 45 |  | Multidrug | NA | NA | 535 | 3368 | n-desmethyltramadol (74.8); o-desmethyltramadol (115.6); eddp (36); ethanol (1.45 g/L); morphine (5.0); cis-tramadol (> 10,000) |
|  |  |  | M | 40 |  | Multidrug | NA | NA | 933 | 4762 | amitriptiline (111.5); chlorprotixene (8.8); eddp (54.3); ethanol (0.25); 7-hydroxyquetiapine (15.1); lidocaine (11.8); nortriptiline (131.2); quetiapine (1,855.0) |
|  |  |  | M | 18 |  | Multidrug | NA | NA | 704 | >10000 | 7-aminoclonazepam (79.2); cetirizine (100.0); n-desmethyltramadol (2.7); o-desmethyltramadol (2.7); eddp (130.5); hydroxizine (35.4); 11-nor-9-carboxy-delta-9-THC (18.6); delta-9-THC (1.2); cis-tramadol (7.7) |
|  |  |  | M | 51 |  | Methadone intoxication | NA | NA | 289 | 4310 | eddp (24.7); ethanol (0.31 g/L) |
|  |  |  | M | 29 |  | Multidrug | NA | NA | 151 | NA | eddp (13.2); lidocaine (134.2); mda (36.2);mdma (565.5); 11-nor-9-carboxy- delta-9-THC (7.2) |
|  |  |  | M | 40 |  | Multidrug | NA | NA | 2 | NA | 7-aminoclonazepam (4.6); atropine (30.6); carbamazepine (452.1); carbamazepine-10,11-epoxide (74.7); eddp (2.3); diazepam (19.9); lidocaine (211.9); metronidazole (quality analysis); midazolam (8.7); nordiazepam (38.2); oxazepam (4.6); paracetamol (2,048.8);phenobarbital (5,949.7); temazepam (3.5) |
|  |  |  | M | 20 |  | Multidrug | NA | NA | 560 | >10000 | alprazolam (8.6); 7-aminoclonazepam (135.0); diazepam (42.4); eddp (54.9); nordiazepam (43.7);temazepam (1.2); delta-9-THC (3.6); 11-nor-9-carboxy- delta-9-THC (31.3); 11-hydroxy- tetrahydrocannabinol (< loq;< 1 ng/ml); |
|  |  |  | M | 35 |  | Methadone intoxication | NA | NA | 403.7 | NA | eddp (28.3); ondansetron (2.8) |
| Giorgetti A [9] | 2021 | Case report | M | 18 | Italy | Methadone intoxication | NA | NA | 3190 | 1700 | flurazepam (32); mirtazapine (68) |
| Franchi A [43] | 2018 | Case report | M | 28 | France | Multidrug | NA | NA | 341 | NA | cyamemazine (0.0189); diazepam (0.144); nordiazepam (0.258); oxazepam (0.012); temazepam (0.012); zolpidem (0.0054) |
| Argo A [44] | 2017 | Retrospective study (autopsy reports) | M | 37 | Italy | Methadone intoxication | Y | NA | 270 | 1430 | NA |
|  |  |  | M | 42 |  | Methadone intoxication | Y | NA | 610 | 3080 | NA |
|  |  |  | M | 20 |  | Methadone intoxication | Y | NA | 370 | 11300 |  |
|  |  |  | M | 35 |  | Methadone intoxication | Y | NA | 890 | 1180 |  |
|  |  |  | M | 35 |  | Methadone intoxication | Y | NA | 720 | 570 |  |
|  |  |  | F | 25 |  | Methadone intoxication | Y | NA | 900 | 700 |  |
|  |  |  | M | 39 |  | Methadone intoxication | Y | NA | 580 | 2000 |  |
|  |  |  | F | 19 |  | Methadone intoxication | Y | NA | 270 | 990 |  |
|  |  |  | F | 25 |  | Methadone intoxication | Y | NA | 380 | 2780 |  |
|  |  |  | M | 25 |  | Methadone intoxication | Y | NA | 530 | 13700 |  |
| Kintz P [45] | 2017 | Case report | M | 4 | France | Methadone intoxication | NA | NA | 354 | NA |  |
| Paul ABM [46] | 2017 | Case report | F | 0 |  | Methadone intoxication | NA | NA | 32 | NA |  |
|  |  |  | F | 1 |  | Methadone intoxication | NA | NA | 440 | NA |  |
|  |  |  | F | 1 |  | Methadone intoxication | NA | NA | 190 | NA |  |
| Bonsignore A [47] | 2016 | Case report | NA | 3 | Italy | Methadone intoxication | N |  | 633 | 5250 |  |
| Madadi P [48] | 2016 | Case report | M | 0 | Canada | Methadone intoxication | Y (mother) | 65 | 79 | NA |  |
|  |  |  | M | 0 |  | Multidrug | Y (mother) | 85-115 | 26 | NA |  |
| Cohen MC [49] | 2025 | Retrospective study | M | 0 | United Kingdom | Methadone intoxication | NA | NA | 64 | NA |  |
|  |  |  | M | 0 |  | Multidrug | NA | NA | 842 | NA | Promethazine (217) |
|  |  |  | F | 0 |  | Methadone intoxication | NA | NA | 109 | NA |  |
| Vignali C [50] | 2015 | Retrospective study (autopsy reports) | M | 39 | Italy | Methadone intoxication | Y | 40-50-50 | 470 | 4700 | delorazepam (30) |
|  |  |  | M | 27 |  | Methadone intoxication | Y | 40-40-40 | 210 | 1380 | diazepam (50); nordiazepam (160) |
|  |  |  | F | 39 |  | Methadone intoxication | Y | NA | 1380 | NA | 7-aminoclonazepam (390); lorazepam (80); venlafaxine (350) |
|  |  |  | M | 25 |  | Methadone intoxication | Y | 30-50-70 | 1080 | 3900 | citalopram (140) |
|  |  |  | M | 22 |  | Multidrug | NA | NA | 520 | 870 | benzoylecgonine (1110); cocaine (60) |
|  |  |  | F | 40 |  | Methadone intoxication | NA | NA | 490 | 1760 | quetiapine (260); vanlafaxine (730) |
|  |  |  | M | 33 |  | Methadone intoxication | NA | NA | 210 | 120 | NA |
|  |  |  | M | 32 |  | Methadone intoxication | NA | NA | 620 | 12700 | NA |
|  |  |  | M | 28 |  | Methadone intoxication | NA | NA | 390 | 2560 | NA |
|  |  |  | M | 38 |  | Methadone intoxication | NA | NA | 3150 | 13600 | NA |
|  |  |  | M | 29 |  | Methadone intoxication | NA | NA | 1460 | 720 | NA |
|  |  |  | F | 16 |  | Methadone intoxication | NA | NA | 690 | 13800 | benzoylecgonine (210) |
|  |  |  | M | 41 |  | Methadone intoxication | NA | NA | 640 | 2370 | mirtazapine (170) |
|  |  |  | M | 26 |  | Methadone intoxication | NA | NA | 520 | 4450 | benzoylecgonine (870) |
|  |  |  | F | 39 |  | Methadone intoxication | NA | NA | 980 | NA | lorazepam (20) |
|  |  |  | M | 39 |  | Methadone intoxication | Y | 40 | 340 | 23000 | NA |
|  |  |  | M | 36 |  | Methadone intoxication | Y | 13 | 480 | NA | NA |
|  |  |  | M | 43 |  | Multidrug | NA | NA | 1300 | 9500 | citalopram (490); levomepromazine (400) |
|  |  |  | M | 24 |  | Methadone intoxication | Y | 40 | 500 | 9520 | benzoylecgonine (190) |
|  |  |  | F | 54 |  | Methadone intoxication | Y | 80 | 980 | NA | NA |
|  |  |  | F | 49 |  | Methadone intoxication | Y | 40 | 3370 | NA | desalkylflurazepam (100) |
|  |  |  | M | 42 |  | Methadone intoxication | Y | 40 | 1060 | NA | promazine (810) |
|  |  |  | M | 33 |  | Methadone intoxication | NA | NA | 970 | 12100 | NA |
|  |  |  | M | 34 |  | Multidrug | NA | NA | 920 | NA | benzoylecgonine (1130);citalopram (310); cocaine (500) |
|  |  |  | M | 26 |  | Multidrug | NA | NA | 590 | 900 | benzoylecgonine (180); cocaine (30);morphine (20) |
|  |  |  | M | 30 |  | Multidrug | NA | NA | 630 | 340 | venlafaxine (980) |
|  |  |  | M | 42 |  | Multidrug | Y | 80 | 380 | 5540 | delorazepam (40); levomepromazine (250); nordiazepam (40); prometazine (70) |
|  |  |  | M | 29 |  | Methadone intoxication | Y | 30 | 130 | 1300 | benzoylecgonine (110) |
|  |  |  | F | 40 |  | Methadone intoxication | NA | NA | 1090 | NA | NA |
|  |  |  | M | 44 |  | Methadone intoxication | NA | NA | 1190 | 7420 | citalopram (350) |
|  |  |  | M | 43 |  | Methadone intoxication | NA | NA | 1500 | NA | benzoylecgonine (1160) |
|  |  |  | M | 45 |  | Multidrug | NA | NA | 2050 | 5120 | sertraline (2000) |
|  |  |  | F | 45 |  | Methadone intoxication | NA | NA | 1270 | 3630 | diazepam (150); nordiazepam (180) |
|  |  |  | M | 33 |  | Methadone intoxication | NA | NA | 1380 | 13900 | benzoylecgonine (480); levomepromazine (90) |
| Iwersen-Bergmann S [51] | 2014 | Retrospective study (autopsy reports) | M | 26 | Germany | Multidrug | Y | NA | 260 | NA | diazepam (100); nordiazepam (100) |
|  |  |  | M | 34 |  | Multidrug | Y | NA | 350 | NA | codeine (5); morphine (71) |
|  |  |  | F | 17 |  | Multidrug | Y | NA | 350 | NA | benzodiazepines (1240); cocaine (20); codeine (20); morphine (110) |
|  |  |  | M | 26 |  | Multidrug | Y | NA | 400 | NA | diazepam (50); morphine (27); nordiazepam (400); oxazepam (200) |
|  |  |  | M | 42 |  | Multidrug | Y | NA | 580 | NA | diazepam (30); morphine (13); nordiazepam (900); oxazepam (40) |
|  |  |  | F | 40 |  | Multidrug | Y | NA | 300 | NA | codeine (120); diazepam (100); lorazepam (300); morphine (14); nordiazepam (600); oxazepam (100); promethazine (90); trimipramine (100) |
|  |  |  | M | 60 |  | Multidrug | Y | NA | 210 | NA | chlorprothixene (100); diazepam (200); nordiazepam (300); oxazepam (500) |
|  |  |  | F | 24 |  | Multidrug | Y | NA | 100 | NA | amphetamine (110); doxepin (90); mdma (70) |
|  |  |  | M | 37 |  | Multidrug | Y | NA | 2500 | NA | diazepam (100); nordiazepam (300); tramadol (2000); venlafaxine (300) |
|  |  |  | F | 29 |  | Multidrug | Y | NA | 370 | NA | bupropione (0.2); doxepin (150); melperone (100); quetiapine (400) |
|  |  |  | M | 51 |  | Multidrug | Y | NA | 410 | NA | codeine (18); diazepam (400); morphine (19); nordiazepam (140) |
|  |  |  | M | 43 |  | Multidrug | Y | NA | 1400 | NA | diazepam (50); thca (18) |
|  |  |  | F | 38 |  | Multidrug | Y | NA | 220 | NA | benzodiazepines (880); cocaine (320); codeine (7); levomepromazine (700); morphine (64) |
|  |  |  | M | 33 |  | Methadone intoxication | Y | NA | 3130 | NA | diazepam (230); nordiazepam (350) |
|  |  |  | F | 19 |  | Multidrug | Y | NA | 740 | NA | benzodiazepines (20); thca (18); thc (2); thc-oh (1) |
|  |  |  | M | 23 |  | Multidrug | Y | NA | 310 | NA | thca (8); thc (1); thc-oh (1) |
|  |  |  | F | 24 |  | Methadone intoxication | Y | NA | 570 | NA | NA |
|  |  |  | M | 49 |  | Methadone intoxication | Y | NA | 560 | NA | NA |
|  |  |  | M | 40 |  | Multidrug | Y | NA | 670 | NA | benzodiazepines (960); diazepam (100); nordiazepam (150) |
|  |  |  | M | 48 |  | Multidrug | Y | NA | 330 | NA | diazepam (200);doxepin (150) |
|  |  |  | M | 36 |  | Methadone intoxication | Y | NA | 310 | NA | NA |
|  |  |  | M | 25 |  | Multidrug | Y | NA | 390 | NA | amphetamine (30); diazepam (400); nordiazepam (130) |
|  |  |  | M | 29 |  | Methadone intoxication | Y | NA | 380 | NA | NA |
|  |  |  | M | 19 |  | Multidrug | Y | NA | 530 | NA | diazepam (30); mirtazapine (100); nordiazepam (80) |
|  |  |  | F | 22 |  | Multidrug | Y | NA | 790 | NA | benzodiazepines (50) |
|  |  |  | M | 40 |  | Multidrug | Y | NA | 550 | NA | diazepam (100); nordiazepam (200); thca (34) |
|  |  |  | M | 46 |  | Multidrug | Y | NA | 990 | NA | benzodiazepines (50) |
|  |  |  | M | 45 |  | Methadone intoxication | Y | NA | 11580 | NA | diazepam (200); nordiazepam (300) |
|  |  |  | M | 57 |  | Multidrug | Y | NA | 920 | NA | benzodiazepines (280); cocaine (50); doxepin (100) |
|  |  |  | M | 45 |  | Multidrug | Y | NA | 300 | NA | diazepam (30); doxepin (100); nordiazepam (200) |
|  |  |  | F | 38 |  | Multidrug | Y | NA | 300 | NA | benzodiazepines (1600); cocaine (60); doxepin (3000) |
|  |  |  | M | 27 |  | Multidrug | Y | NA | 60 | NA | diazepam (380); nordiazepam (340) |
|  |  |  | M | 36 |  | Multidrug | Y | NA | 140 | NA | ethanol (0.87 g/L) |
|  |  |  | M | 49 |  | Multidrug | Y | NA | 60 | NA | ethanol (2.29 g/L) |
|  |  |  | M | 27 |  | Multidrug | Y | NA | 350 | NA | buprenorphine (4); diazepam (90); doxepin (90); norbuprenorphine (2); nordiazepam (100) |
|  |  |  | F | 52 |  | Multidrug | Y | NA | 470 | NA | ethanol (1.86 g/L); nortrimipramine (300); tripipramine (1470) |
|  |  |  | M | 32 |  | Multidrug | Y | NA | 1080 | NA | benzoylecgonine (1900); citalopram (110); cocaine (230);diazepam (100); methylecgonine (580);nordiazepam (100); tramadol (7300) |
| Tournel G [52] | 2014 | Case report | m | 1 | France | Methadone Intoxication | NA | NA | 73 | 1784 |  |
| Corliss RF [53] | 2013 | Case report | M | 23 | United States | Multidrug | Y | 40 | 154 | NA | alprazolam (14.1); delta-9-THC l (5.9) |
| Kupiec TC [54] | 2011 | Case report | M | 23 | United States | Methadone intoxication | N | NA | 510 | NA | Hydroxyzine (540000)  Trichloroethanol (8300) |
| Letsky MC [55] | 2011 | Case report | F | 51 | United States | Multidrug | Y | 720 | 570 | NA | ethanol (0.013 g/L); doxylamine (22); oxycodone (17) |
| Palmiere C [56] | 2011 | Case report | M | 40 | United States | Methadone intoxication | NA | NA | 290 | 160 | ethanol (1.06 g/L) |
| Mistry V [57] | 2010 | Case report | M | 0 | United Kingdom | Multidrug | N | A | 277 | NA |  |
|  |  |  | M | 1 |  | Methadone intoxication | N |  | 298 | NA | sertraline (472);  delta-9-THC (1.3) |
| Carson HJ [58] | 2009 | Case report | M | 25 | United States | Multidrug | NA | NA | 450 | NA | norsertraline (2817); sertraline (472); delta-9-THC (1.3) |
| Kobek M [59] | 2009 | Case report | M | 0 | Poland | Methadone intoxication | Y (mother) | NA | 240 | 310 |  |
| Sidlo J [60] | 2009 | Case report | M | 1 | Slovakia | Methadone intoxication | NA | NA | 700 | 3200 |  |
| Fahey T [61] | 2003 | Case report | M | 39 | Namibia | Multidrug | Y | 60 | 510 | NA | amitriptyline (500); nortriptyline (80); temazepam (1080) |
| Grass H [62] | 2003 | Retrospective study |  |  | Germany | Methadone intoxication | Y | NA | 660 | NA |  |
|  |  |  |  |  |  | Methadone intoxication | Y | NA | 440 | NA | ethanol (0.03 g/L) |
|  |  |  |  |  |  | Multidrug | Y | NA | 200 | NA | ethanol (0.01 g/L); bromazepam (370) |
|  |  |  |  |  |  | Multidrug | Y | NA | 210 | NA | ethanol (1.37 g/L); delta-9-THC (2); diazepam (380); doxepin (420) |
|  |  |  |  |  |  | Multidrug | Y | NA | 220 | NA | ethanol (0.69 g/L); morphine (580); diazepam (190); amitryptiline (11000); sulpirid (6400) |
|  |  |  |  |  |  | Multidrug | Y | NA | 210 | NA | ethanol (1.22 g/L); doxepin (6700); carbamazepine (12200) |
|  |  |  |  |  |  | Multidrug | Y | NA | 260 | NA | ethanol (0.79 g/L); delta-9-THC (43); diazepam (100) |
|  |  |  |  |  |  | Multidrug | N |  | 1580 | NA | ethanol (2.36 g/L); morphine (30); diazepam (190) |
|  |  |  |  |  |  | Multidrug | N |  | 620 | NA | ethanol (0.03 g/L); diazepam (690) |
|  |  |  |  |  |  | Multidrug | N |  | 430 | NA | ethanol (1.62 g/L); diazepam (420); |
|  |  |  |  |  |  | Multidrug | N |  | 50 | NA | amphetamine; morphine (100) |
|  |  |  |  |  |  | Multidrug | N |  | 50 | NA | ethanol (0.01gL); morphine (290); MDMA (920) |
|  |  |  |  |  |  | Multidrug | N |  | 50 | NA | amphetamine (60); ethanol (0.01 g/L); imiptamine (170); delta-9-THC (2) |
| Langlois NE [63] | 2002 | Case report | M | 0 | Australia | Multidrug | NA | NA | 900 | NA | paracetamol (32000); phenobarbitone (1400) |
| Li L [64] | 2000 | Case report | M | 4 | United States | Methadone intoxication | Y | 40 | 600 | NA |  |
|  |  |  | F | 5 |  | Methadone intoxication | NA | NA | 600 | NA |  |
| Perret G [36] | 2000 | Retrospective study (autopsy reports) | F | 27 | Switzerland | Methadone intoxication | Y | 180 | 1400 | NA | benzodiazepines; cocaine |
|  |  |  | M | 34 |  | Methadone intoxication | Y | 45 | 500 | NA | carbamazepine |
|  |  |  | M | 40 |  | Methadone intoxication | Y | 120 | 1900 | NA | benzodiazepines |
|  |  |  | M | 21 |  | Multidrug | Y | 80 | 800 | NA | flunitrazepam (700) |
|  |  |  | M | 38 |  | Methadone intoxication | Y | 80 | 1400 | NA |  |
|  |  |  | M | 38 |  | Methadone intoxication | NA | 80 | 1400 | NA |  |

**Table S3.** Cases from the University of Bologna. MMT: methadone maintenance therapy. NA: Not Available. Y: Yes. M: Male. F: Female.

| **Case Id** | **Sex** | **Age** | **Cause of death** | **MMT** | **Posology**  **(Mg/Die)** | **Methadone Blood Concentration (Ng/Ml)** | **Concomitants**  **(ng/ml In Blood)** |
| --- | --- | --- | --- | --- | --- | --- | --- |
| 1 | M | 35 | Multidrug | Y | 45 | 700 | cocaine (2265); venlafaxine (569) |
| 2 | M | 31 | Multidrug | NA | NA | 329 | cocaine (4588.6); morphine (51.7) |
| 3 | M | 42 | Multidrug | Y | 50 | 503 | cocaine (138); paroxetine (43); trazodone (291);aripiprazole (52) |
| 4 | M | NA | Methadone intoxication | NA | NA | 343 | NA |
| 5 | M | 30 | Multidrug | Y | NA | 149 | nordiazepam (115); tramadol (383); zolpidem (17) |
| 6 | M | 57 | Multidrug | NA | NA | 1057 | alprazolam (33.2); lorazepam (90.3) |
| 7 | M | 35 | Multidrug | Y | NA | 298 | cocaine (34.6); benzoylecgonine (1346.8); ethanol (0.44 g/L) |
| 8 | M | 46 | Multidrug | NA | NA | 321 | mirtazapine (165.8), diazepam and nordiazepam (traces) |
| 9 | M | 56 | Multidrug | Y | NA | 506 | cocaine (137); benzoylecgonine (160); diazepam (999); nordiazepam (910) |
| 10 | F | 30 | Multidrug | Y | 90 | 1112 | THC (2.1), 11-OH-THC (1.7), diazepam (60.5), nordiazepam (78.5), mirtazapine (11.4) |
| 11 | M | 58 | Multidrug | NA | NA | 527 | morphine (581.954); codeine (88.415) |
| 12 | M | 43 | Multidrug | NA | NA | 78 | nordiazepam (144.70); diazepam (33.70);oxazepam (49.8);temazepam (23.20); morphine (169.2) |
| 13 | M | 44 | Multidrug | NA | NA | 80 | cocaine (1380); morphine (582); codeine (43) |
| 14 | M | 41 | Pure methadone | NA | Y | NA | NA |
| 15 | F | 20 | Multidrug | NA | NA | >2 | morphine (993); codeine (34); cocaine (36); alprazolam (129); trazodone (247) |
| 16 | F | 32 | Multidrug | NA | NA | 1679 | diazepam (40); mitrazapine (186.6); clorpromazine (60.2); lorazepam (64.8); nordiazepam (53.5); cocaine (6393) |
| 17 | M | 58 | Multidrug | NA | NA | 1057 | alprazolam (33.2); lorazepam (90.3) |
| 18 | M | NA | Multidrug | NA | NA | 106 | morphine (1481.3); codeine (78.7); 6-monoacethylmorphine (14.8); heroin (traces); ethanol (1.83 g/L) |
| 19 | F | 40 | Multidrug | NA | NA | 660 | bromazepam (10.2); alprazolam (2.4); cocaine (15.8) |
| 20 | M | 44 | Trauma | NA | NA | 41 | ethanol (1.95 g/L); cocaine (31.4) |
| 21 | M | 40 | Trauma |  |  | 35 | levomepromazine (36.7); quetiapine (91.3); 7-aminoclonazepam (54.1); norquetiapine (224.4); valproic acid (4.8 mcg/ml), cocaine (105), morphine (14); codeine (30.3); amphetamine (24.0); THC (3.0) |
| 22 | M | 46 | Multidrug | Y | NA | 272 | mophine (594.1); codeine (42.5) |
| 23 | M | 42 | Multidrug | Y | NA | 503 | paroxetine (42.8); trazodone (291.2); aripiprazole (52.4); dehydroaripiprazole (15.4); cocaine (137.7); cocaethylene (43.3) |
| 24 | M | 35 | Multidrug | NA | NA | 442 | diazepam (traces); mirtazapine (5.9); promazine (44.2); tramadol (traces) |
| 25 | F | 31 | Trauma | Y | NA | 1112 | diazepam (60.5); mirtazapine (11.4); nordiazepam (78.5); THC (2.1); 11-OH-THC (1.7) |
| 26 | F | 47 | Trauma | NA | NA | 120 | diazepam and nordiazepam (traces); fluoxetine (3235.5); olanzapine (957.1); clotiapine (traces); desmethylolanzapine (25.9);trazodone (107.2) |
| 27 | M | 30 | Multidrug | NA | NA | 1065 | ethanol (0.67 g/L); diazepam (187); temazepam (traces); citalopram (265.2 ng/L); trazodone (107.2), olanzapine (255.5 ng/ml); nordiazepam (192); morphine (19) |
| 28 | M | 31 | Multidrug | NA | NA | 343 | ethanol (0.20 g/L) |
| 29 | M | 35 | Multidrug | NA | NA | 256 | diazepam (15.9); venlafaxine (569.6), o-desmethylvenlafaxine (194.3), cocaine (2265) |
| 30 | M | 56 | Multidrug | NA | NA | 506 | diazepam (999); nordiazepam (910); cocaine (137) |
| 31 | M | 30 | Multidrug | NA | NA | 149 | diazepam (traces); quetiapine (traces); nordiazepam (115); tramadol (383); zolpidem (17); |
| 32 | M | 52 | Multidrug | Y | NA | 130 | midazolam (804); alpha-OH-midazolam (126.2); morphine (1540) |
| 33 | M | 38 | Multidrug | NA | NA | 80 | cocaine (1351); morphine (277.4), codeine (23.2); 6-mam (200.3) |
| 34 | M | 26 | Multidrug | NA | NA | 906 | THC (traces) |
| 35 | M | 64 | Multidrug | NA | NA | 228 | ethanol (0.52 g/L); promethazine (traces); loxapine (traces); fluphenazine (traces);cocaine (1271.6);morphine (417.2); codeine (269) |
| 36 | M | 29 | Multidrug | NA | NA | 469 | delorazepam (870.2), clotiapine (980) |

**Table S4.** Methadone blood concentration (ng/ml) distribution based on gender, MMT, and methodone intoxication/multidrug intoxication

|  | min | 1st quartile | median | mean | 3rd quartile | max |
| --- | --- | --- | --- | --- | --- | --- |
| Males | 2.3 | 310.0 | 502.9 | 772.8 | 923.1 | 11580.0 |
| Females | 100.0 | 335.8 | 570.0 | 767.5 | 1062.5 | 3370.0 |

Wilcoxon Mann-Withney test: W = 3405, p-value = 0.3458

| MMT yes | 60.0 | 310.0 | 502.9 | 802.2 | 892.5 | 11580.0 |
| --- | --- | --- | --- | --- | --- | --- |
| MMT no | 181.0 | 381.8 | 474.5 | 754.9 | 1120.5 | 1830.0 |

Wilcoxon Mann-Withney test: W = 804.5, p-value = 0.6601

| Multidrug | 2.3 | 262.5 | 399.0 | 524.2 | 667.5 | 2500.0 |
| --- | --- | --- | --- | --- | --- | --- |
| Methadone intoxication | 130.0 | 404.7 | 630.0 | 994.3 | 1147.8 | 11580.0 |

Wilcoxon Mann-Withney test: W = 6167, p-value = 1.066e-05

**Table S5**. Results of the linear regression analysis interpolating methadone blood concentrations with drugs that have potential pharmacokinetic/pharmacodynamic interactions with methadone. CNS depressants: Central Nervous System depressants. CYP3A4 inhibitors: Cytochrome P450 3A4 inhibitors. CYP3A4 inducers: Cytochrome P450 3A4 inducers. CYP2B6 inducers: Cytochrome P450 2B6 inducers.

| Variable | Coefficient | p-value |
| --- | --- | --- |
| Intercept | 929.71 | <0.001 |
| CNS depressants | -213.97 | 0.175 |
| CYP3A4 inhibitors | -445.74 | 0.661 |
| QT prolongers | -48.56 | 0.777 |
| CYP3A4 inducers | -344.90 | 0.503 |
| CYP2B6 inducers | - | - |


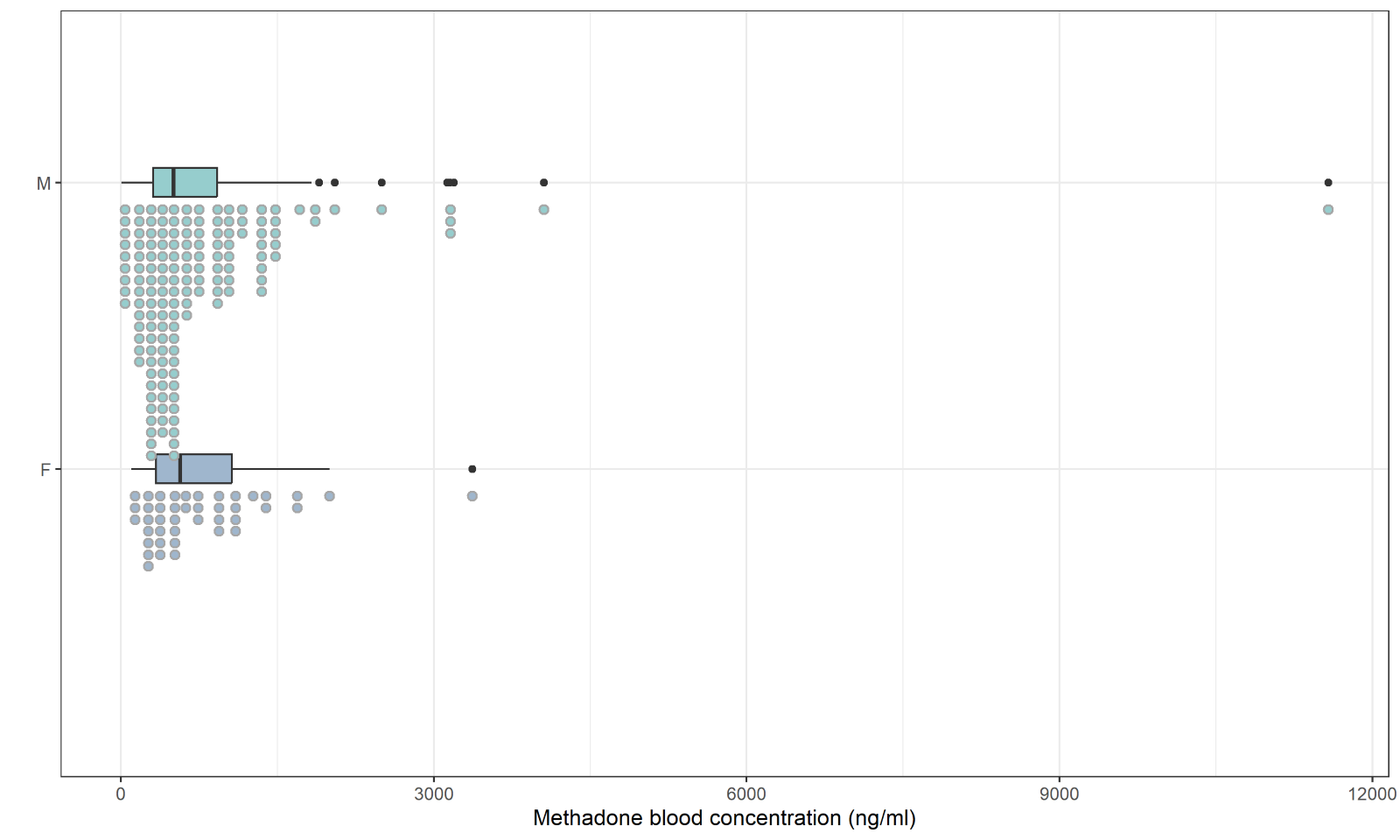

**Figure S1**. Methadone blood concentration (ng/ml) in male and female subjects


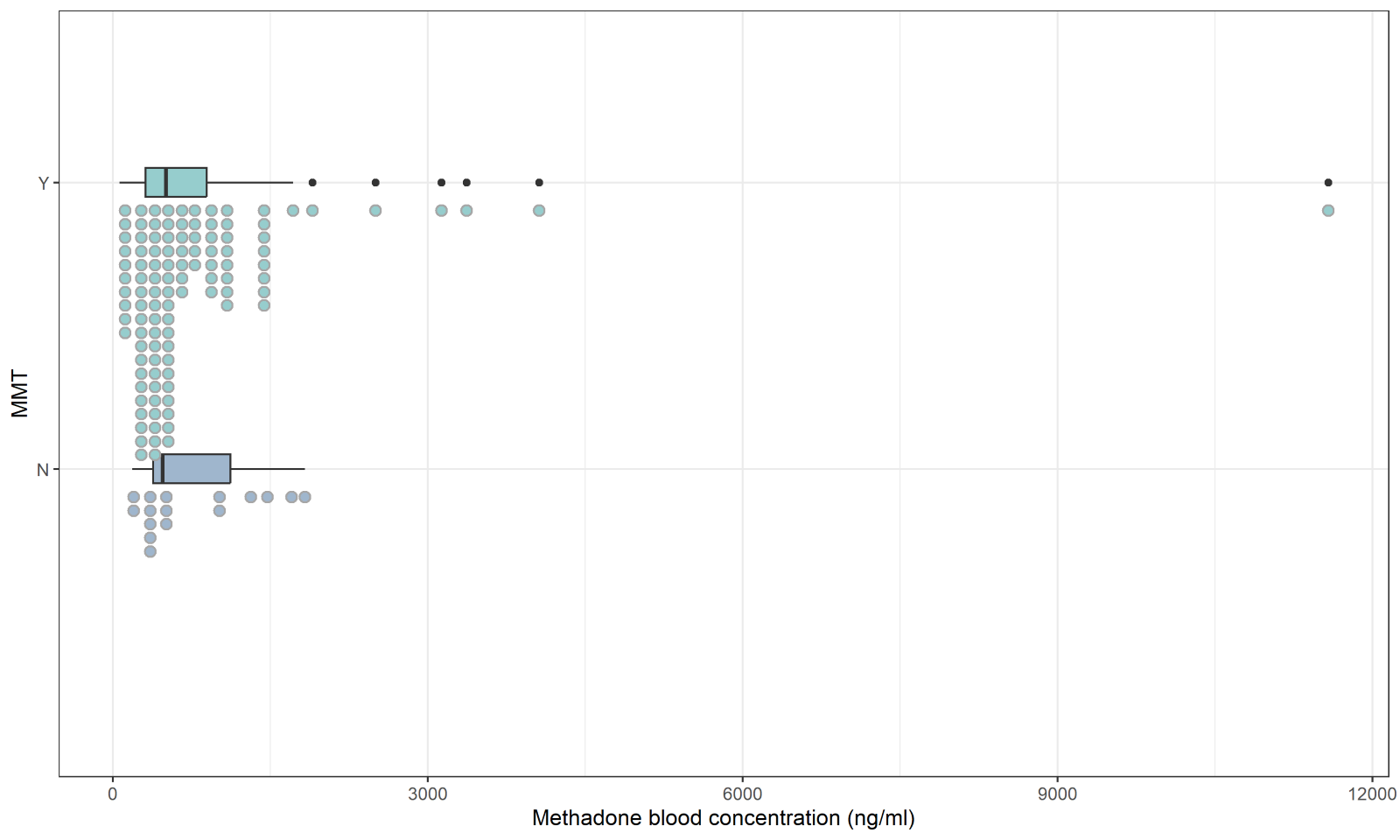

**Figure S2.** Methadone blood concentration (ng/ml) in deceased with and without MMT (Methadone Maintanance Treatment).


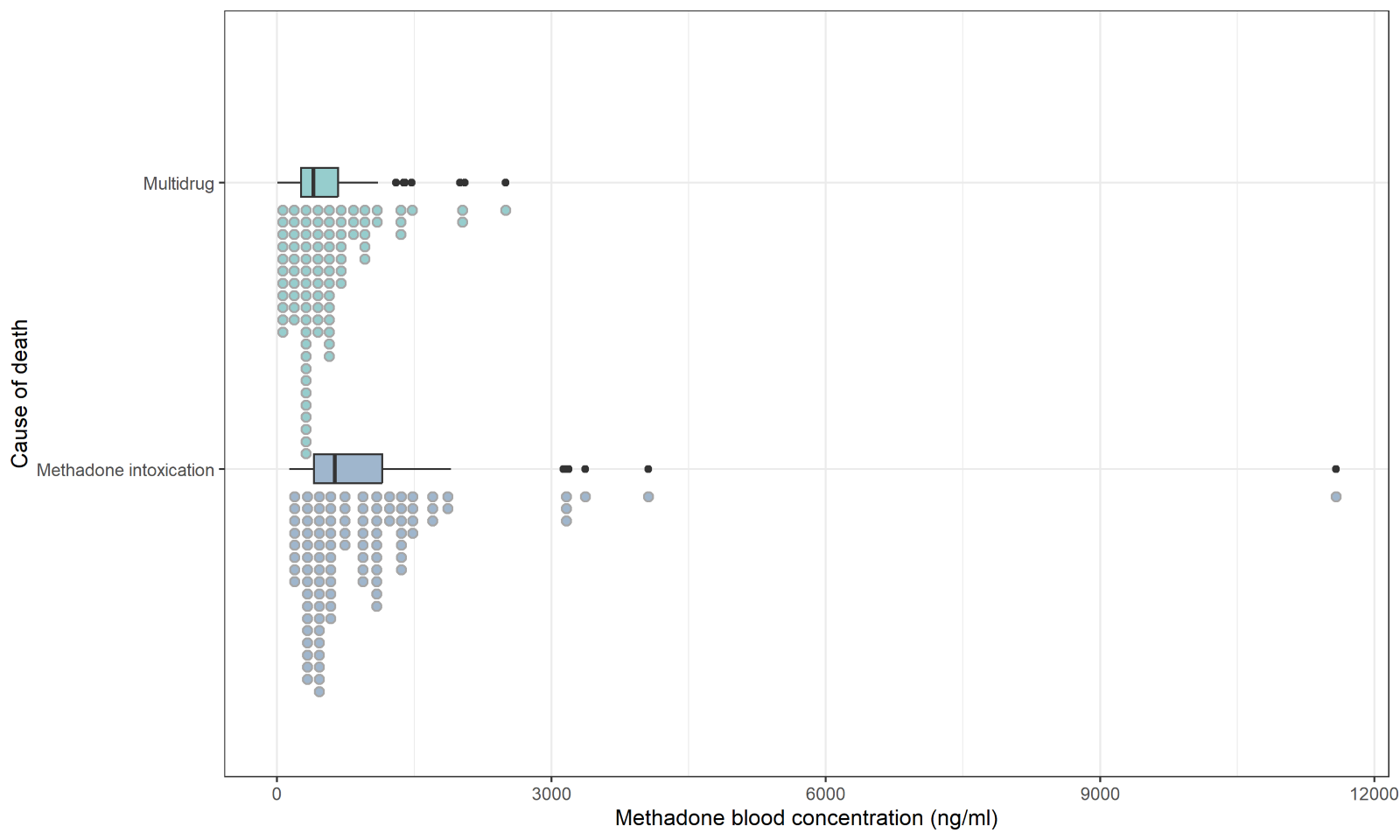

**Figure S3.** methadone blood concentration (ng/ml) in methadone intoxication and multidrug intoxication**.**
